# Effects of polyimide curing on image sticking behaviors of flexible displays

**DOI:** 10.1038/s41598-021-01364-6

**Published:** 2021-11-08

**Authors:** Hyojung Kim, Jongwoo Park, Sora Bak, Jungmin Park, Changwoo Byun, Changyong Oh, Bo Sung Kim, Chanhee Han, Jongmin Yoo, Dongbhin Kim, Jangkun Song, Pyungho Choi, Byoungdeog Choi

**Affiliations:** 1 Technology Reliability Team, OLED Business, Samsung Display Co., Ltd., Asan, South Korea; 2 Department of Semiconductor and Display Engineering, Sungkyunkwan University, Suwon, South Korea; 3 Research Center for Materials, Components and Equipment, Advanced Institutes of Convergence Technology (AICT), Seoul National University, Suwon, South Korea; 4 Department of Applied Physics, Korea University, Sejong, South Korea; 5 Department of Electrical and Computer Engineering, Sungkyunkwan University, Suwon, South Korea

**Keywords:** Electrical and electronic engineering, Electronics, photonics and device physics

## Abstract

Flexible displays on a polyimide (PI) substrate are widely regarded as a promising next-generation display technology due to their versatility in various applications. Among other bendable materials used as display panel substrates, PI is especially suitable for flexible displays for its high glass transition temperature and low coefficient of thermal expansion. PI cured under various temperatures (260 °C, 360 °C, and 460 °C) was implemented in metal–insulator–metal (MIM) capacitors, amorphous indium gallium zinc oxide (a-IGZO) thin-film transistors (TFT), and actual display panels to analyze device stability and panel product characteristics. Through electrical analysis of the MIM capacitor, it was confirmed that the charging effect in the PI substrates intensified as the PI curing temperature increased. The threshold voltage shift (ΔV_th_) of the samples was found to increase with rising curing temperature under negative bias temperature stress (NBTS) due to the charging effect. Our analyses also show that increasing ΔV_th_ exacerbates the image sticking phenomenon observed in display panels. These findings ultimately present a direct correlation between the curing temperature of polyimide substrates and the panel image sticking phenomenon, which could provide an insight into the improvement of future PI-substrate-based displays.

## Introduction

Flexible displays are displays typically fabricated on a plastic substrate. They are considered as state-of-the-art display technology since they enable unprecedented applications in various industries including clothing, automobiles, and even artificial intelligence^1^. Unlike displays made on fixed glass substrates, flexible displays use polymer substrates for their flexibility and stretchability. Although various polymer materials have been studied as substrate materials, polyimide (PI), a material well known for its high heat resistance and low coefficient of thermal expansion, is mainly used^2,3^. PI films with fluorinated groups are widely used because of their excellent optical transparency, low absorptivity/emissivity, and high thermal conductivity^4,5^. In general, to fabricate an organic light emitting diode (OLED) flexible display on a PI substrate, a thin-film transistor (TFT) is first deposited as a driving device, and then an OLED is deposited on it as a light emitting device. As OLEDs are very vulnerable to oxidation and moisture, SiO_2_ or Al_2_O_3_ is deposited on the PI substrate as a barrier layer before TFT deposition to prevent penetration of oxygen and moisture^6^. Since the glass transition temperature of PI is less than 500 °C, a low-temperature process is essential to the fabrication of PI-substrate-based TFTs^7^. As such, low-temperature polycrystalline silicon (LTPS) TFTs with good mobility or amorphous indium gallium zinc oxide (a-IGZO) TFTs with a good on/off ratio are used^8–11^. However, the occurrence of specific threshold voltage (V_th_) behavior during the reliability evaluation of TFTs fabricated on PI substrates was recently reported^12,13^. In a display panel, several TFTs are closely interconnected to emit light from a single OLED pixel, and if an abnormal behavior occurs in the V_th_ of a specific TFT, a difference in luminance among OLED pixels occurs. In our last study, we found that this specific behavior of V_th_ is exhibited in the TFT fabricated on a PI substrate under negative bias temperature stress (NBTS). We also confirmed that when NBTS is applied to the TFT, fluorine ions (F^−^) can be generated in the PI, at which time the interface between the PI and the barrier becomes charged and the V_th_ of the TFT fabricated thereon can shift abnormally^14^.

As described above, PI is mainly used as a substrate for flexible displays, and to investigate whether the properties of display substrates have a distinguishable effect on the V_th_ shift anomaly in the TFTs, we manufactured and analyzed devices and products fabricated on PI substrates for three different curing temperatures (260 °C, 360 °C, and 460 °C). Metal–insulator–metal (MIM) capacitors, a-IGZO TFTs, and display panels were utilized to investigate the material properties of PI, the device stability, and the product characteristics, respectively. Fourier transform infrared (FT-IR) analysis on single-layer PI film shows no significant difference in curing rate of PI even under different curing temperatures. However, the V_th_ shift of the TFTs fabricated on the PI substrates was discovered to increase under NBTS with rising temperature of the PI curing processes. Through secondary-ion mass spectrometry (SIMS) analysis of MIM capacitors before and after NBTS, we confirmed that the cause of the abnormal V_th_ shift lies in the increasing amount of F^−^ accumulating in the upper part of PI as the curing temperature increases. Therefore, it is inferred that higher PI curing temperature leads to greater fluorine ion-induced charging effect in PI. Image sticking evaluation was carried out with actual display panels to investigate the effect of PI curing temperature on product-level display panels. Image sticking, which refers to a phenomenon in which the previous image remains visible on the screen in the next frame, is an essential quality evaluation index used in the display industry. The image sticking evaluations we performed indicate that the image sticking phenomenon worsened in the panels of higher PI curing temperatures. In this study, we confirmed that the curing temperature of PI is correlated with the image sticking issue and investigated the cause of this relationship through chemical, electrical, and physical analysis methods.

## Results

### Analysis of PI properties on curing temperature

**Figure 1:**
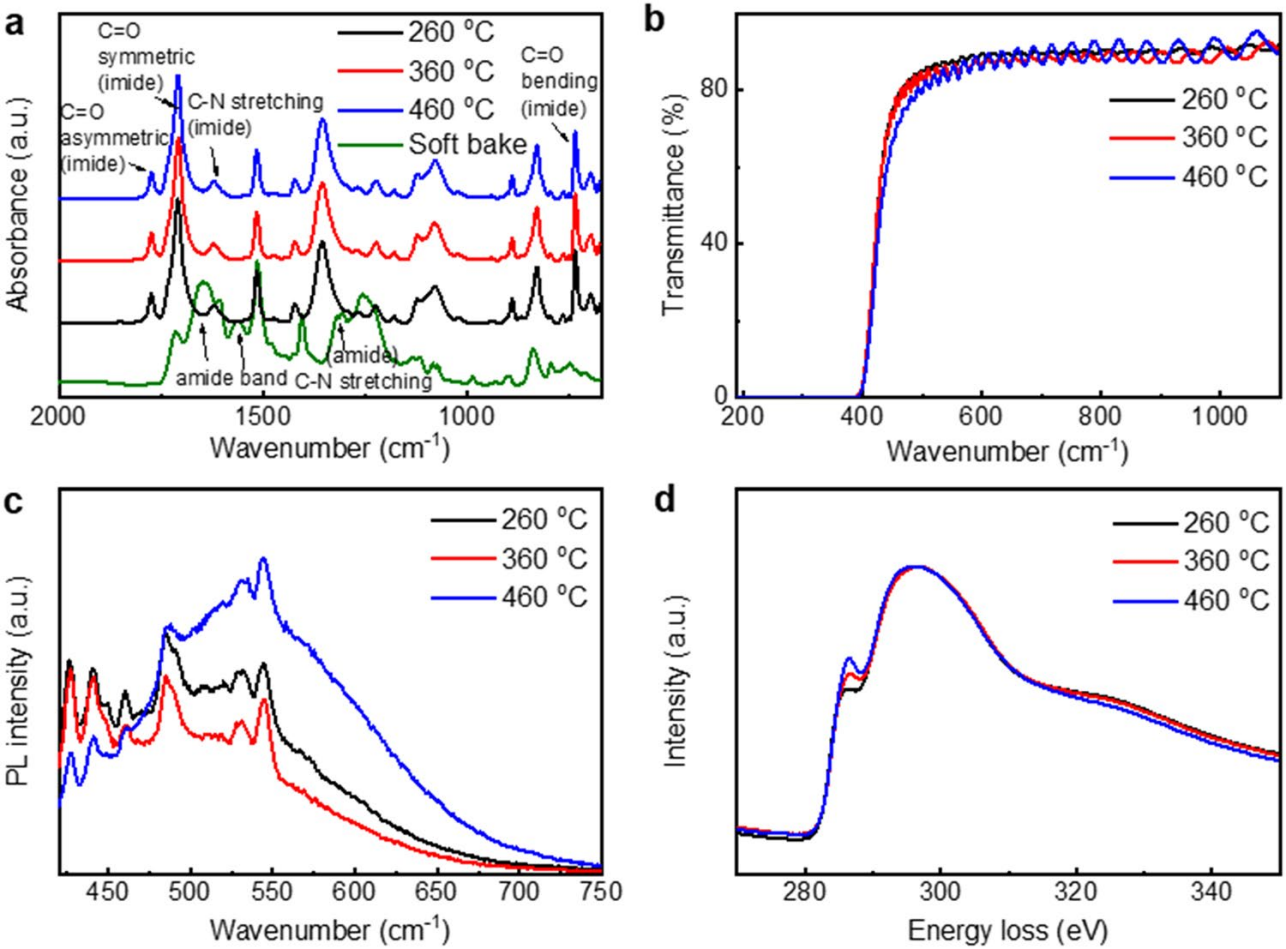
Characterization of polyimide materials by curing temperature. (**a**) FT-IR spectra of soft-baked PAA at 80 °C and cured PI at different temperatures. (**b**) UV–vis spectra, (**c**) PL spectra excited at 400 nm, and (**d**) EELS spectra of PI depending on curing temperature.

Spin-coated polyamic acid (PAA) was baked at 80 °C for 30 min to remove the solvent, then cured in two steps under inert conditions: first at 260 °C for 30 min and then at 260 °C, 360 °C, or 460 °C for 4.5 h, respectively. FT-IR can be used to identify the degree of curing. The spectra did not exhibit any amide bonds, as shown in Fig. 1a. This finding indicates that temperatures above 260 °C are suitable to ensure full imidization of PAA^15,16^. There was no significant difference in the roughness of PI under different curing conditions (refer to Supplementary Fig. S1).

The inter- and intra-chain charge transfer (CT) mainly affected the optical, mechanical, physical, and electrical properties of the aromatic PI^17^. When the CT is increased, light absorption is also enhanced but photoluminescence (PL) yield is weakened^18,19^. The UV–vis spectra of PI films with different curing temperatures are shown in Fig. 1b. Even though the band gap is identical as 3.1 eV based on the Tauc plot, obviously the PI cured at 460 °C has the lowest transmittance among the three samples. The transmittance in 450–500 nm is reduced as the curing temperature of PI is increased, which means an increase in the charge transfer properties. Figure 1c and Supplementary Fig. S2 show the PL spectra of PI depending on the curing temperature. PL is a valuable parameter to analyze the charge transfer complex (CTC) properties because the PL intensity decreases when the CTC properties are enhanced^20^. The PL intensity in the high-wavelength range (above 500 nm) decreases as the curing temperature increases from 260 to 360 °C. A higher curing temperature corresponds to an enhanced molecular packing^21^. Moreover, molecular aggregation causes PL quenching, especially in the low-energy range, which induces interchain CT^17,22,23^. At temperatures more than 460 °C, the PL intensity increases and decreases in the high and low wavelength ranges, respectively. Generally, a higher curing temperature induces molecular aggregation with interchain CT and hydrogen bonding and quenching of the PL properties of PI, as observed in the case of samples cured at 260 °C and 360 °C. However, at temperatures more than 400 °C, interchain interactions transform from interchain CT to dipole–dipole interactions, resulting in an increased PL intensity at high wavelengths. Consequently, PI cured at 460 °C exhibits a different trend^24^.

To confirm the change in carbon backbone of the PI, electron energy loss spectroscopy (EELS) analysis was carried out. Figure 1d shows that the distribution of sp^3^ and sp^2^ carbons within the PI backbone varies depending on the curing temperature. It is well known from previous studies that the presence of the π^∗^ peak at 285 eV in all the carbon samples is characteristic for π bonds in sp^2^ coordinated carbon^25^. The amount of graphitic sp^2^ carbon increases under elevating temperature as indicated in Fig. 1d. As shown in the FT-IR results, it can be concluded that curing is complete over 260 ℃. Thus, temperature treatments above 260 ℃ do not increase the curing rate, but the conductivity increases as the amount of sp^2^ carbon increases inside the PI backbone. Consistent with the above PL study results, it can be seen that the higher the heat treatment temperature, the greater the increase in internal C = C bond in PI backbone and molecular packing.

### MIM capacitor stability

**Figure 2:**
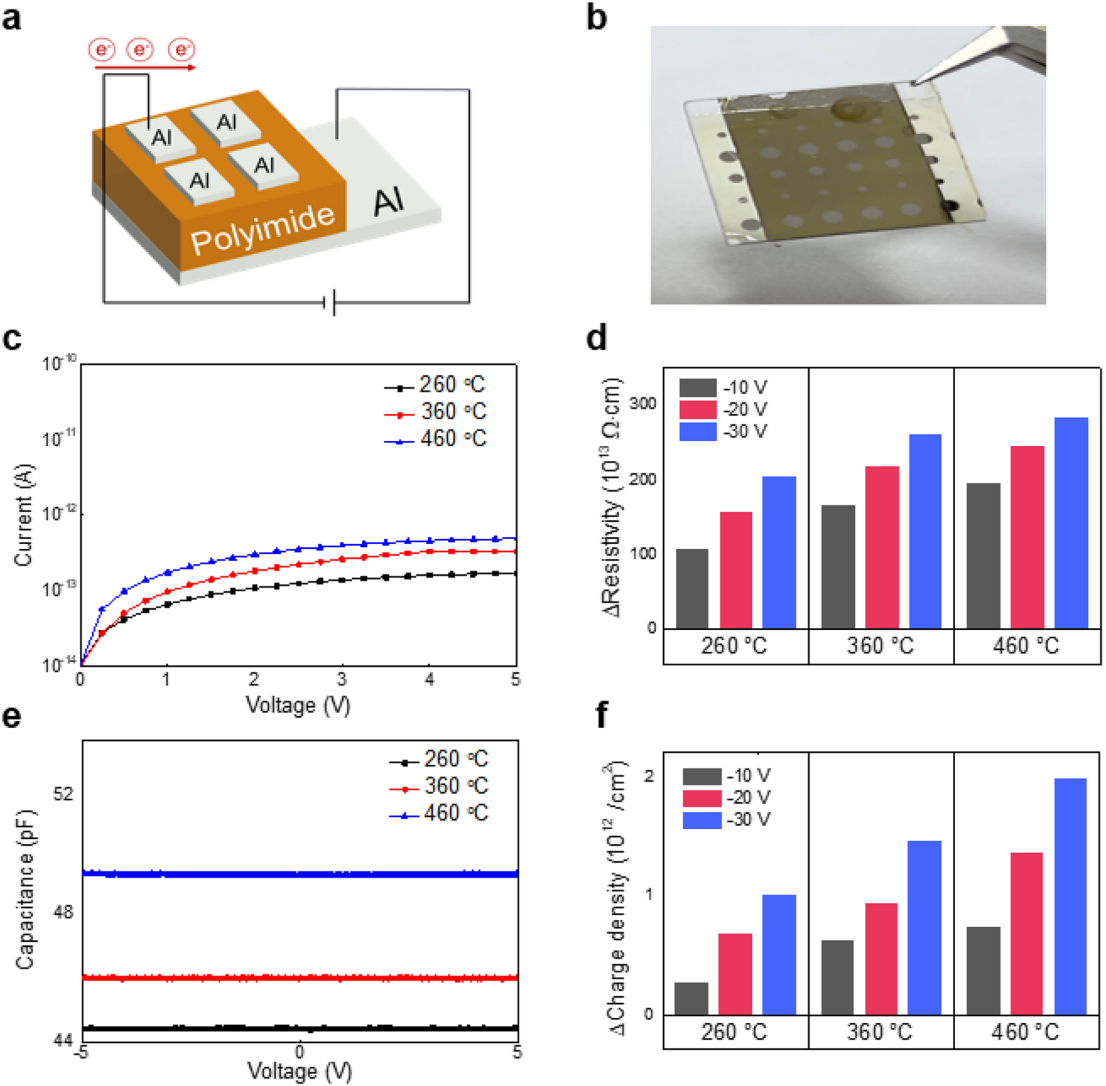
Electrical characteristics of MIM capacitors. (**a**) Structure of MIM capacitor (Al/PI(260 °C)/Al, Al/PI(360 °C)/Al, Al/PI(460 °C)/Al). (**b**) Actual MIM capacitor used for measurement. (**c**) Initial I–V plot of MIM capacitors. (**d**) Change in resistivity of MIM capacitors under NBTS. (**e**) Initial C–V plot of MIM capacitors. (**f**) Change in charge density of MIM capacitors under NBTS.

To conduct an electrical analysis of PI according to the curing temperature, a MIM capacitor was fabricated using PI as an insulator, and an electrical measurement was performed. Figure 2a, b present the schematic structure of the MIM capacitor and a picture of the device. Figure 2c–e shows the current–voltage (I–V) and capacitance–voltage (C–V) initial characteristics of MIM capacitors. Through the initial I–V and C–V measurements of the MIM capacitors, we extracted the initial parameters of PI according to the curing temperature (refer to Supplementary Table S1). After curing for each PI temperature, the thickness was determined through secondary electron microscopy (SEM) analysis. The thicknesses of PI films cured at 260 ℃, 360 ℃, and 460 ℃ were 5.1 µm, 4.9 µm, and 4.6 µm, respectively (refer to Supplementary Fig. S3). With increasing curing temperature, the molecular density is increased, which induces the reduction in the thickness of polyimide^21^. The volume resistivity was extracted from the I–V plot of the MIM capacitor, and the dielectric constant was extracted through the C–V measurement (refer to Supplementary Table S1).

Figure 2c shows that the volume resistivity of the PI film decreased as the curing temperature increased. This agrees well with the previous material analysis results, which shows that as the curing temperature is increased, the volume resistivity is decreased because the C = C bonding inside the PI backbone and molecular packing are increased. Figure 2d is a plot showing the change in volume resistivity of MIM capacitors after NBTS at PI curing temperatures of 260 °C, 360 °C and 460 °C. At 70 ℃, stress voltages of − 10 V, − 20 V, and − 30 V were applied to the top electrode for 1 h to confirm the change in volume resistivity. The change in leakage current was the greatest in the PI cured at 460 ℃ (refer to Supplementary Fig. S4). The application of NBTS leads to the increase in leakage current because the mobile charge of PI acts as a defect source^26,27^. In addition, since the conductivity increases due to increased C=C bonding and molecular packing as the curing temperature increases, the change in leakage current increases. Figure 2f shows the change in the charge density of the MIM capacitor after NBTS. PI cured at 460 °C showed the largest change in the charge density according to the applied stress voltage. The change of PI charge density induced an electric field. The electric field that can affect the TFT active layer fabricated on the PI substrate was extracted by converting the capacitance change of the PI film into electric charge (refer to Supplementary Figs. S5a–c). Supplementary Fig. S5e shows a dependence of capacitance on frequency over a 100 Hz to 100 kHz range. It can be seen that, in that range, the capacitance is constant. These results represent that there is no significant difference in the polarity behavior by dipolar polarization for each PI curing temperature. The PI charging phenomenon can cause a change in capacitance and can be linked to the V_th_ shift of TFTs fabricated on a PI substrate^14^.

### Physical analysis result of MIM capacitor before/after NBTS

**Figure 3:**
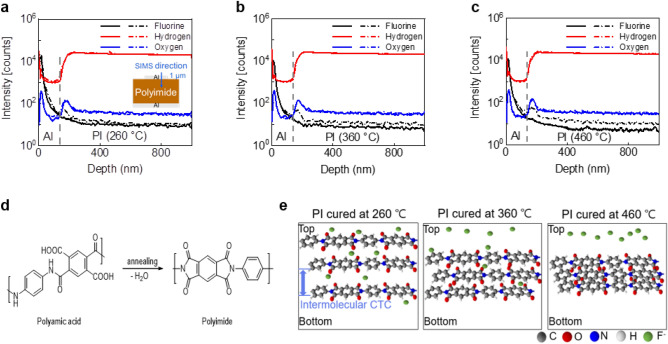
SIMS analysis and PI structure. (**a**–**c**) SIMS analysis of MIM capacitors before (dashed line) and after (dash dotted line) NBTS. (**d**) Chemical structure of PI. (**e**) Geometry of polyimide by curing temperature.

To determine the origin of the cause of the capacitance change of MIM capacitors after NBTS, we performed SIMS, a common physical analysis method. Figure 3a–c shows SIMS analysis data of MIM capacitors before/after NBTS. First, H^−^ and O^−^ before and after NBTS were analyzed to confirm whether there was an effect from oxygen or moisture permeation from the outside. All three capacitors showed no difference before/after NBTS. However, it was confirmed that the accumulated amount of F^−^ in the vicinity of the upper electrode increased after NBTS compared to the initial measurement. F^−^ in the insulator, which acts as a mobile charge, affects the device characteristics^28,29^. In terms of TFTs, the accumulated F^−^ at the PI interface can cause a negative charging effect and V_th_ shift. Although it is difficult to quantitatively compare the increases, it was confirmed that the amount of F^−^ accumulated in the upper electrodes before/after NBTS increased as the PI curing temperature increased. The cause of this phenomenon can be inferred from the previous chemical analysis of Fig. 1. The chemical structure of aromatic PI, which is composed of aromatic dianhydride and diamine, is shown in Fig. 3d. As the curing temperature increases, the conductivity of the PI film is increased by the higher molecular packing density and charge transfer complex, which in turn enhances the mobility of F^−^ in the PI film. Under NBTS, the higher the curing temperature of PI, the more F^−^ moves to the vicinity of the upper electrode. This mechanism is schematically shown in Fig. 3e.

### Electrical characteristics of a-IGZO TFTs

**Figure 4:**
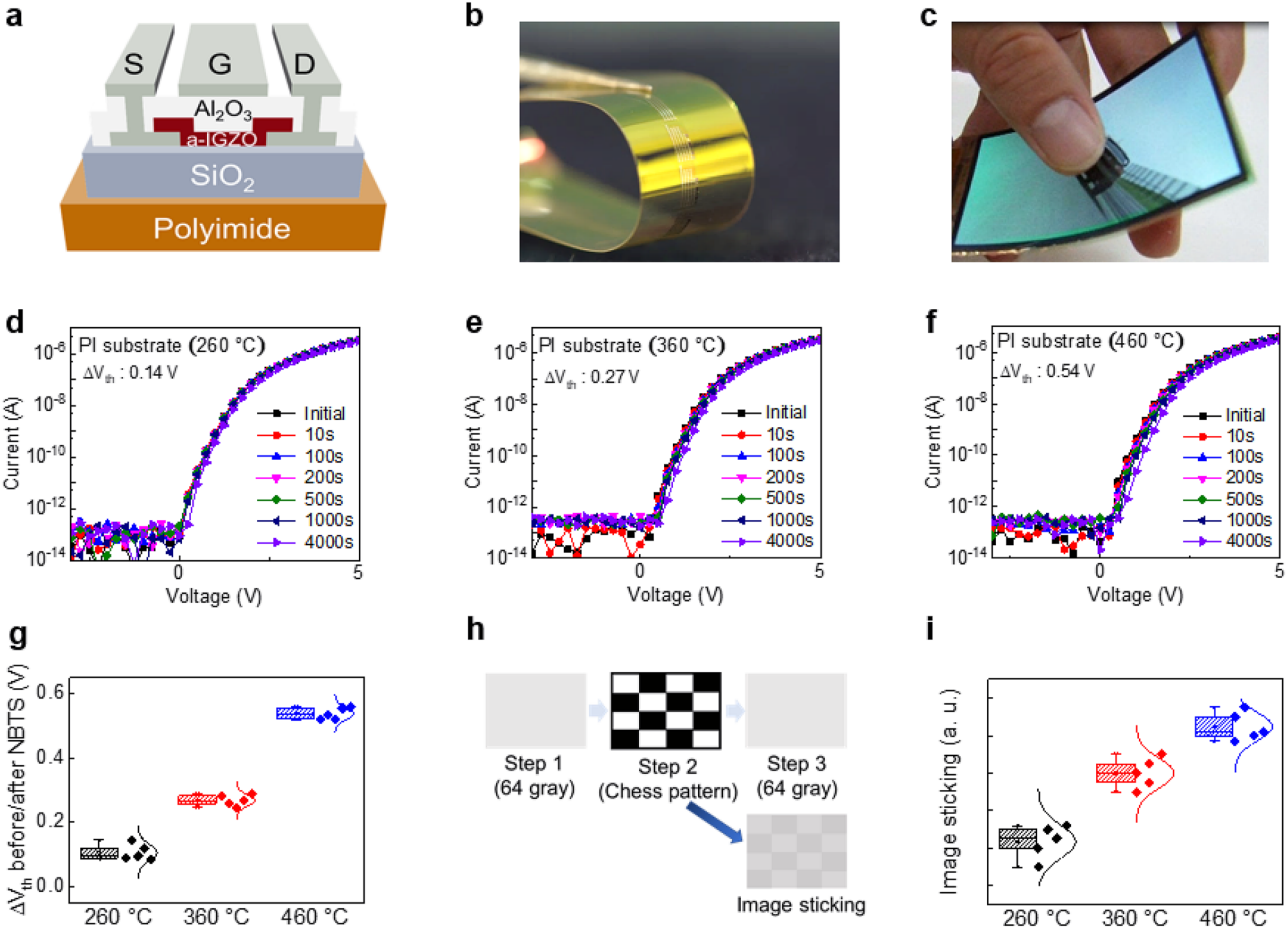
Electrical properties of single a-IGZO TFTs and OLED panel characteristics. (**a**) Schematic structure of a-IGZO TFT used in the experiment. (**b**) A picture of the panel manufactured on the PI substrate. (**c**) A picture of the fabricated flexible displays. (**d**) I_D_–V_G_ plot under NBTS of TFT fabricated on PI substrate cured at 260 °C. (**e**) I_D_–V_G_ plot under NBTS of TFT fabricated on PI substrate cured at 360 °C. (**f**) I_D_–V_G_ plot under NBTS of TFT fabricated on PI substrate cured at 460 °C. (**g**) ΔV_th_ after NBTS for 5 samples per TFTs. (**h**) Panel image sticking evaluation method and image sticking phenomenon. (**i**) Image sticking results of panels manufactured on substrates with different PI curing temperatures.

a-IGZO TFTs were fabricated on PI substrates with curing temperatures identical to those of PI used in the aforementioned MIM capacitors. The schematic structure of the TFT used in this study is shown in Fig. 4a, b is a picture of the panel manufactured on the PI substrate, and Fig. 4c is a picture of the actual fabricated flexible displays. NBTS was performed to confirm the difference in stability of the three sets of TFTs with PI curing temperatures of 260 °C, 360 °C, and 460 °C. Figure 4d–f shows the changes of transfer characteristics over time for the TFTs under NBTS. A stress voltage of − 10 V was applied to the gate at 70 °C for 4000 s. The initial and post-NBTS parameters of the three types of TFTs with different substrate curing temperatures are summarized in Supplementary Table S2. All the TFTs confirmed the positive shift of V_th_ after NBTS. In general, it is well known that in a-IGZO TFTs under NBTS holes become trapped in the gate insulator, resulting in V_th_ shift in the negative direction^30^. We reported in our previous paper the abnormal V_th_ behaviors during the reliability evaluation and its cause for the TFTs fabricated on PI substrates showing V_th_ shift in the positive direction even under the NBTS condition^13^. This is further clarified in this study as the results indicate the active hole carriers in the a-IGZO film are trapped in the barrier layer because the electric field induced by the accumulated F^−^ at the PI and barrier interface is greater than the negative field applied to the TFT gate (refer to Supplementary Fig. S5d). Fluorine ions accumulated at the PI and barrier interface form electronic polarization when a negative field is applied to the TFT gate, causing a charging effect^31^. Due to this mechanism, the electrons accumulate in the bottom side of the active layer and increase the turn-on voltage of a-IGZO TFT. Figure 4g shows the box plot of the amount of V_th_ shift after NBTS with four samples for each device.

The TFTs fabricated on the PI substrate cured at 260 °C, 360 °C, and 460 °C had positive shifts of 0.14 V, 0.27 V, and 0.54 V in V_th_ after NBTS, respectively. It can be seen that the amount of positive shift of V_th_ is larger for the TFTs fabricated on PI substrates cured at higher temperatures due to the fact that the higher curing temperatures of PI lead to a greater amount of F^−^ accumulation at the PI and barrier interface after NBTS, ultimately causing the charging effect.

### Image sticking results

Evaluation of the image sticking phenomenon was performed with flexible OLED panels consisting of the aforementioned a-IGZO TFTs. One of the indicators that can evaluate the quality of the display panel is the display image sticking method^11^. Figure 4h is the schematic illustration of the chess image sticking evaluation method used in this experiment. We measured the luminance at the first 64 gray pattern, which was then aged for a certain time under the pattern where the black/white pattern intersects, and then measured the luminance again at the 64 gray pattern. The time at which the luminance measured after aging exceeds a certain level compared to that of the initial 64 gray pattern is defined as an image sticking time value (refer to Supplementary Fig. S6). As shown in Fig. 4h, in the panel manufactured on the PI substrate, the luminance of the part that had been aged in the black pattern was decreased. This can be attributed to the V_th_ shift of a specific TFT in the OLED pixel driving circuit. The uniformity of V_th_ of TFTs is critical because the OLED display controls the luminance via the current of the driving TFT. However, in the mass-production of display products, LTPS or a-IGZO TFTs are manufactured on a large-area substrate, so it is difficult to control V_th_ uniformity. Thus, as shown in Supplementary Fig. S6d, a V_th_ compensation circuit is used to maintain a constant current flowing through the OLED, even if the V_th_ of the driving TFT is different^32^. When the panel has a black pattern, NBTS is applied to the switching transistor in charge of the diode connection of the actual compensation circuit. If the V_th_ of the switching transistor in the compensation circuit is positively shifted, the luminance may decrease in the panel (refer to Supplementary Fig. S6e). As a result, it can be seen that the cause of the decrease in luminance in the black pattern is the V_th_ positive shift of the switching transistor in the compensation circuit. Figure 4i is the evaluation result of the image sticking of the panel in which TFTs with different PI curing temperatures were fabricated with a backplane. It is evident that the image sticking of the display panel using a TFT with a low PI curing temperature as the backplane is small. In the TFT devices we have evaluated so far, we verified the origin of positive V_th_ shift and its correlation with the PI curing temperature. It was also confirmed that the positive V_th_ shift of TFTs that occurs during NBTS can cause image sticking on the panel.

## Discussion

In this study, physical property analysis and electrical analysis were conducted by varying the curing temperature of PI, which is used as a substrate for flexible displays. It was confirmed that the curing rate of PI was the same for all samples and that molecular packing, molecular aggregation, and inter-chain CT increased with rising curing temperature. Through the electrical analysis, it was shown that the resistivity decreased as the PI curing temperature increased, which increased the mobility of F^−^ in the PI when stress was applied, causing large amounts of F^−^ accumulation in the PI. This accumulated F^−^ causes a V_th_ shift of the a-IGZO TFT fabricated on the PI substrate. In addition, this study clarified that this phenomenon is the cause of image sticking in display products. Because PI has excellent thermal properties and high transparency, it has been studied as a substrate for flexible devices in many fields as well as flexible displays. However, it was revealed in this study that the stability of devices and products manufactured on PI substrates may vary depending on the PI curing temperature. These results are expected to help improve the stabilities of PI-based flexible devices in the future.

## Methods

### Device fabrication

In this study, TFTs with three structures were used. Figure 1a shows a schematic cross section of the top-gate-staggered a-IGZO TFT used in the experiment. The substrate was imidized through heat treatment after spin coating on glass using PAA. The PAA was soft baked at 80 °C for 30 min to remove the solvent, and the curing temperature was split into 260 °C, 360 °C, and 460 °C with a given curing time of 4.5 h. The following processes after the deposition of the PI were the same for all TFTs. A 100 nm thick SiO_2_ used as the barrier layer was deposited using plasma enhanced chemical vapor deposition. A Mo layer was deposited onto the barrier layer and then patterned via photolithography to form the source and drain electrodes. A 50 nm thick active layer of a-IGZO was formed using DC sputtering at room temperature. A 100 nm thick Al_2_O_3_ layer as a gate insulator was deposited via atomic layer deposition. Contact holes were formed on the gate insulator by photolithography and dry etching. The gate electrode was deposited using DC sputtering. The Mo (50 nm) gate electrode was deposited with DC sputtering and patterned to form the gate electrode. To activate the a-IGZO channel layer, annealing was performed under vacuum at 300 °C for 1 h after finishing all processes. Three TFTs with different PI substrate curing temperatures were used in the experiment. In addition, Al/PI/Al MIM capacitors for each PI curing temperature were used in the experiment to conduct electrical analyses before/after stress.

### Electrical characterization

I–V and C–V measurements were performed with an Agilent B1500A Semiconductor Analyzer and Keysight E4980A LCR meter to confirm the initial electrical characteristics of each MIM capacitor and TFT. To determine the difference in stability of MIM capacitors, capacitance and leakage current changes were confirmed by applying − 10 V, − 20 V, and − 30 V bias stresses to the top electrode at 70 °C with 1-h intervals. For NBTS evaluation, − 10 V gate voltage was applied to the a-IGZO TFTs at 70 °C for 4000 s, and the results confirmed the changes in electrical characteristics.

## Supplementary Information

Supplementary Information 1.

## Data Availability

All data related to this paper can be requested from the corresponding author upon reasonable request.
