# Supplementary material for: Effects of polyimide curing on image sticking behaviors of flexible displays
Source: Sci Rep. 2021 Nov 8;11:21805. doi: 10.1038/s41598-021-01364-6 (PMC8575959; doi:10.1038/s41598-021-01364-6)
Supplement: Supplementary file 1 — Supplementary Information 1. [file 41598_2021_1364_MOESM1_ESM.docx]

***Supplementary information***

**Effects of polyimide curing on image sticking behaviors of flexible displays**

Hyojung Kim, Jongwoo Park, Sora Bak, Changwoo Byun, Changyong Oh, Bo Sung Kim, Chanhee Han, Jongmin Yoo, Jungmin Park, Dongbhin Kim, Jangkun Song, Pyungho Choi, and Byoungdeog Choi*

H.J. Kim, Dr. J.W. Park, Dr. S.R. Bak

Technology Reliability Team, OLED Business Samsung Display Co., Ltd., 181 Samsung-ro, Tangjeong-myeon, Asan-si 31454, Republic of Korea

H.J. Kim, J.M. Park

Department of Semiconductor and Display Engineering, Sungkyunkwan University, Cheoncheon-dong 300, Jangan-gu, Suwon-si 16419, Republic of Korea

Dr. B.C. Byun

Research Center for Materials, Components and Equipment, Advanced Institutes of Convergence Technology (AICT), Seoul National University, 145 Gwanggo-ro, Yeongtong-gu, Suwon-si, Gyeonggi-do 16229, Republic of Korea

Prof. B.S. Kim, C.Y. Oh

Department of Applied Physics, Korea University, 2511 Sejong-ro, Sejong-si 30019, Republic of Korea

C.H. Han, J.M. Yoo, D.B. Kim, Prof. J.K. Song, Prof. P.H. Choi, Prof. B.D. Choi

Department of Electrical and Computer Engineering, Sungkyunkwan University, Cheoncheon-dong 300, Jangan-gu, Suwon-si 16419, Republic of Korea

E-mail: [bdchoi@skku.edu](mailto:bdchoi@skku.edu)

Keywords: Polyimide; thin-film transistor; flexible display; image sticking; display panel; PI charging


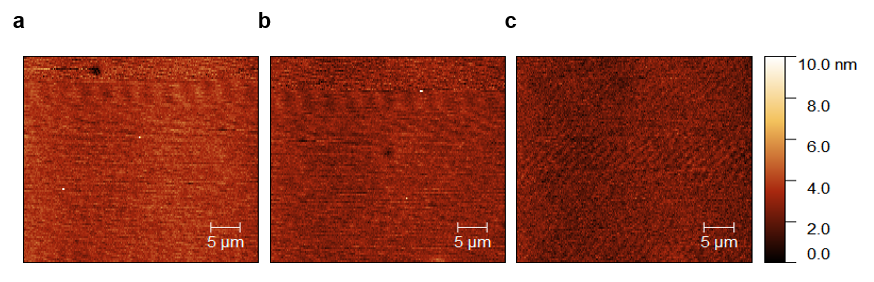


**Supplementary Figure S1.** AFM image of PI films cured at (a) 260 °C, (b) 360 °C, and (c) 460 °C.

(RMS: 0.549 nm, 0.537 nm, and 0.559 nm, respectively.)


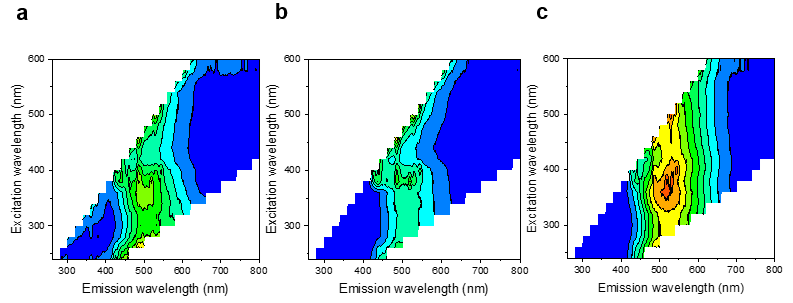


**Supplementary Figure S2.** Excitation and emission maps for photoluminescence from PI films cured at (a) 260 °C, (b) 360 °C, and (c) 460 °C.


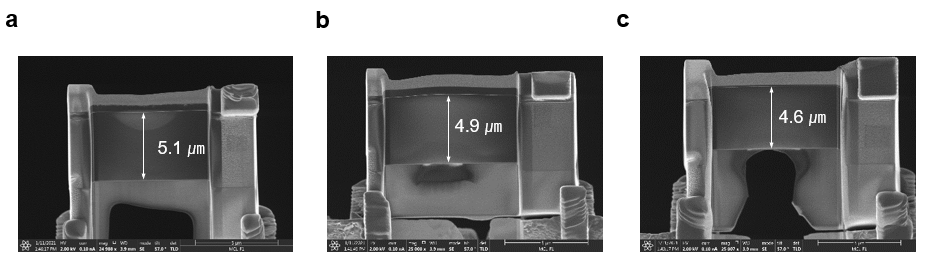


**Supplementary Figure S3.** Thicknesses of PI films measured by SEM for the samples with curing temperature at (a) 260 °C, (b) 360 °C, and (c) 460 °C.


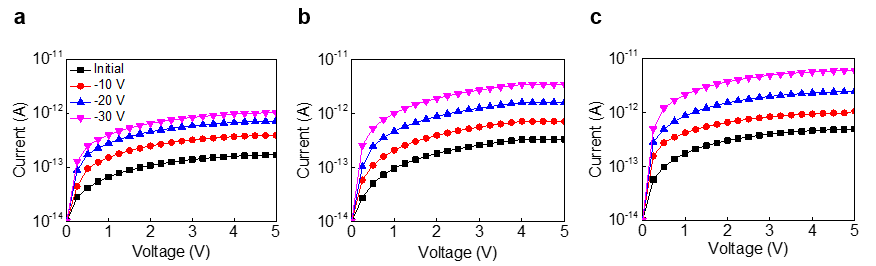


**Supplementary Figure S4.** I–V plots before/after bias temperature stressing of the MIM capacitors: (a) Al/PI (260 °C)/Al, (b) Al/PI (360 °C)/Al, and (c) Al/PI (460 °C)/Al.


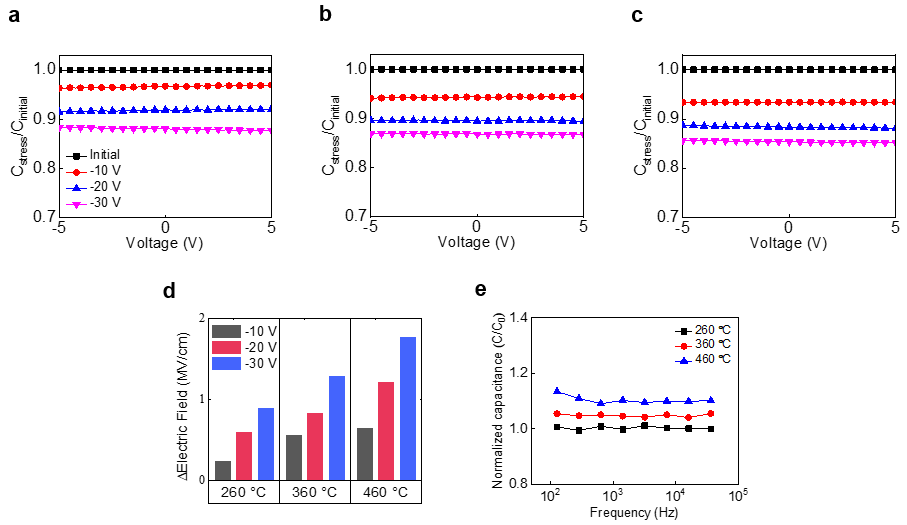


**Supplementary Figure S5.** Bias-temperature-stress-induced normalized capacitance-voltage characteristics; changes in capacitance of metal/PI/metal capacitors cured at (a) 260 °C, (b) 360 °C and (c) 460 °C for 4.5 h. (d) Electric field extracted according to the amount of charge change of the PI capacitor according to voltage applied. (e) Frequency dependence of capacitance for three different PI curing temperatures.

After NBTS, the amount of change in capacitance was obtained through C-V measurements, and the Q value (in Coulombs) was extracted through Q=CV. To confirm whether it can affect the a-IGZO TFT active area fabricated on the actual PI, the extracted charge is converted into a field (E=V/d) that can be applied to the actual active layer in the PI/barrier (SiO_2_ thickness: 100 nm)/a-IGZO structure, and this is shown as a bar graph in Fig. 5(d). Metal/PI/metal normalized capacitance-voltage measurements are important to understand the hysteresis properties of thin film transistors. We investigated the bias stress and frequency-dependent capacitance changes of PI by measuring the voltage sweep from -5 V to 5 V and frequency sweep from 10^2^ to 10^5^ Hz while fixing the curing temperatures to 260 °C, 360 °C and 460 °C. However, the capacitances were not changed for every set of conditions. This means that image sticking due to the threshold voltage shift is attributed not to the device driving conditions but to material changes from the curing conditions.


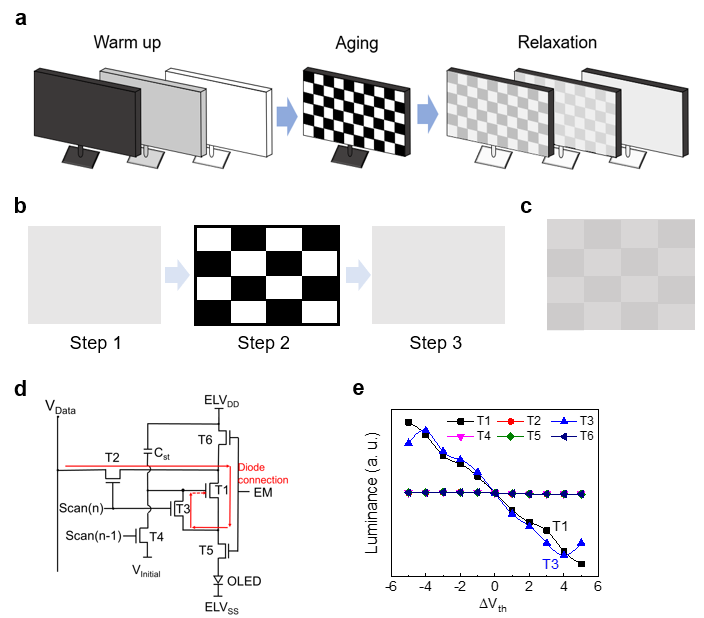


**Supplementary Figure S6.** Image sticking measurement of OLED flexible panel. (a) Image sticking evaluation method. (b) Image sticking evaluation step. (c) Image sticking image. (d) OLED pixel driving circuit. (e) Panel luminance change simulation result according to V_th_ shift of the TFT in the pixel driving circuit.

Figure 6(a) is a general display image sticking evaluation method^1,2^. The first step is a warm-up period to recover the display from possible initial image sticking and ensure a steady state. The second stage is aging. Aging patterns depend on the method used, and depending on the application, environmental conditions such as temperature may also need to be adjusted. After aging, the relaxation period begins. At this stage, the display switches to a relaxed image. After the delay time, the Luminance Measuring Kamera (LMK) performs continuous photometric measurements. Final static image values are evaluated based on these relaxation images. Figure 6(b) is the image sticking evaluation method used in this study. The image sticking time is measured in four steps: the luminance value from the initial 64 Gray is extracted in step 1; then the panel is aged with a chess pattern with black and white patterns in step 2; then the luminance value is extracted again after the panel switches to the 64 Gray; finally, the time when the luminance value in step 3 is measured to be a certain level of the initial luminance value in step 1 is calculated in step 4. The standard for this difference in luminance is the difference in luminance when image sticking cannot be recognized by the human eye^3^. Figure 6(c) shows an image sticking phenomenon that appears in a display manufactured on a polyimide substrate after aging in Fig. 6(b). It was confirmed that the luminance decreased in the black patten aging area. Figure 6(d) is the circuit diagram of the pixel in the panel^4^. It was confirmed whether the luminance difference in the actual panel was caused by changing the V_th_ of the TFTs of pixel parts T1 to T6. Figure 6(e) shows the simulation result confirming whether a difference in luminance occurs in the panel according to the change in V_th_ of the TFT.

**Supplementary Table S1.** Electrical properties of PI film by curing temperature.

|  | 260 ℃ | 360 ℃ | 460 ℃ |
| --- | --- | --- | --- |
| Dielectric constant | 2.87 | 2.85 | 2.83 |
| Volume resistivity (Ω∙cm) | $5.19\times{10}^{15}$ | $2.43\times{10}^{15}$ | $1.99\times{10}^{15}$ |

**Supplementary Table S2.** Electrical properties before and after NBTS of TFTs fabricated on PI films with different curing temperatures.

|  | 260 ℃ | 360 ℃ | 460 ℃ |
| --- | --- | --- | --- |
| V_th_ (V) | 1.42 | 1.58 | 1.69 |
| ΔV_th_ (V) | 0.14 | 0.27 | 0.54 |
| S.S (V/dec) | 0.38 | 0.35 | 0.37 |
| ΔS.S (V/dec) | 0.01 | 0.01 | 0.02 |

ΔV_th_ was extracted using the threshold current method, in which a specific gate voltage with a drain current (I_D_) of $10 nA \times\frac{W}{L}$ under a drain voltage of 5.1 V (saturation region) is measured before (initial) and after (4000 s) NBTS. The ΔV_th_ is then calculated by subtracting the initial V_th_ from the post-stress V_th_.

S.S was extracted through the following formula.

$s.s = \left( \frac{dV_{G}}{d\log I_{D}} \right)_{max}$

**Supplementary References**

1. I. Rostscholl, U. Kruger. Aspects of Image Sticking Evaluations Using Imaging Luminance Measurement Devices. SID Symp. Dig. Tech. **50**, 695-698 (2019).

2. Kim, J. M. et al. An Evaluation Methodology for Display Retention Measurement. SID Symp. Dig. Tech. 49, 80-83 (2018).

3. ICDM, RESIDUAL IMAGE (10.4),“ in ÎDMS: Information Display Measurements Standard, (2012).

4. Chung, H. K. et al. *Organic light emitting device pixel circuit and driving method therefor*. U.S. Patent No 7,414,599 (2008).
